# Investigating the Effect of *Pulicaria jaubertii* as a Natural Feed Additive on the Growth Performance, Blood Biochemistry, Immunological Response, and Cecal Microbiota of Broiler Chickens

**DOI:** 10.3390/ani13061116

**Published:** 2023-03-22

**Authors:** Abdulrahman S. Alharthi, Nawaf W. Alruwaili, Hani H. Al-Baadani, Maged A. Al-Garadi, Ghalia Shamlan, Ibrahim A. Alhidary

**Affiliations:** 1 Department of Animal Production, College of Food and Agriculture Science, King Saud University, P.O. Box 2460, Riyadh 11451, Saudi Arabia; abalharthi@ksu.edu.sa (A.S.A.);; 2 Department of Community Health Sciences, College of Applied Medical Sciences, King Saud University, P.O. Box 10219, Riyadh 11433, Saudi Arabia; 3 Department of Food Science and Nutrition, College of Food and Agriculture Science, King Saud University, P.O. Box 11451, Riyadh 11362, Saudi Arabia

**Keywords:** *Gallus domesticus*, *Pulicaria jaubertii*, performance indicators, pre-inflammatory cytokines, microbiota

## Abstract

**Simple Summary:**

*Pulicaria jaubertii* is considered a medicinal plant traditionally used to treat various human diseases because it contains many biologically active compounds, including monoterpenoids, sesquiterpenoids, diterpenoids, flavonoids, and phenols, which could have beneficial effects on broiler performance and health, although there are no studies on the use of this plant in broilers. The aim of this study was to investigate the effect of *Pulicaria jaubertii* (3–9 g *Pulicaria jaubertii* powder/kg basal diet) on the performance, blood biochemistry, internal organs, gene expression related to immune response, and cecal microbiota of broiler chickens. We conclude that *Pulicaria jaubertii* has a positive effect on overall performance, immune response, and microbiota.

**Abstract:**

Based on the biologically active compounds of *Pulicaria jaubertii* studied so far, there are no studies on the use of this plant in broilers. Therefore, the present study aims is to investigate the effect of *Pulicaria jaubertii* on the performance, blood biochemistry, internal organs, gene expression related to immune response, and the cecal microbiota of broiler chickens. A total of two hundred and forty male broilers were used and divided into four diet groups (T1 = 0, T2 = 3, T3 = 6, and T4 = 9 g *Pulicaria jaubertii* powder/kg basal diet). The performance evaluation, serum biochemical parameters, internal organ indicators, cytokines’ gene expression, and microbiota colonization were determined. The study results showed that this plant was rich in nutrients, some fatty acids, and bioactive phenolic compounds. All growth performance indicators and relative liver weight were improved by *Pulicaria jaubertii* levels (T2 to T4) with no effect on feed intake. T3 and T4 showed higher total protein and lower triglycerides and total cholesterol. Birds fed *Pulicaria jaubertii* showed immune regulation through the modulation of pre-inflammatory cytokines and increased *mucin-2 and secretory Immunoglobulin A* compared with the control group. Diet groups (T2 to T4) had higher quantities of *Lactobacillus* spp. and lower levels of *Salmonella* spp. than the control group. We conclude that *Pulicaria jaubertii* could be used as a feed supplement for broilers due to its beneficial effects on overall performance, immune response, and microbiota. Further studies are recommended to investigate the potential mechanism of *Pulicaria jaubertii* in broilers.

## 1. Introduction

In recent years, several studies have shown that the addition of aromatic medicinal plants as natural feed additives in broiler diets can improve growth performance, health, nutrient digestibility [1,2], innate immunity [3], and antioxidant status [4]. These natural additives depend on the plant part used (e.g., seed, leaf, root, or bark) and the processing technique (e.g., extraction with non-aqueous solvents) to alter the active compounds in the final product. In addition, supplementation with aromatic medicinal plants alters normal gut microflora and modulates immune responses [5]. However, the use of aromatic medicinal plants as growth promoters and antimicrobial agents is becoming increasingly important as an alternative to antibiotics [6]. Due to the development of resistant bacteria, the use of antibiotics in a diet as a subtherapy to improve the performance and health status of broiler chickens has been banned recently, starting with the European Union in 2006 [7,8]. Munyaka et al. [9] reported that the use of aromatic medicinal plants as an alternative to antibiotics in poultry could improve growth performance and immune system integrity. A study by Pirgozliev et al. [2] reported that a commercial medicinal plant mixture improved overall growth performance, nutrient retention, and intestinal cytokine expression in broiler chickens. In addition, Cho et al. [10] indicated that the medicinal plants improved growth performance and inhibited pathogenic bacteria (*Clostridium perfringens* and *Escherichia coli*) in the small intestine of broiler chickens.

Renewed interest in the search for new dietary supplements from natural plant sources has attracted worldwide attention in recent years. *Pulicaria jaubertii*, also known as Anssif or Alkhaw’ah, is an aromatic medicinal plant of the Asteraceae family that is widely distributed in southern Saudi Arabia and Yemen [11]. *Pulicaria jaubertii* is a medicinal plant traditionally used to treat various human diseases, including inflammation, colds, diuretics, and digestive disorders [12,13]. Al-Maqtari et al. [14] and Mohammed et al. [15] showed that *Pulicaria jaubertii* extract has potent antioxidant, antimicrobial, and anti-inflammatory effects. It is also used as a food supplement [16]. According to Hussein et al. [17], food and pharmaceutical industries could use *Pulicaria jaubertii* oil as a safe, natural substitute for synthetic antioxidants. El-Ghaly et al. [18] reported that *Pulicaria jaubertii* extract has antihypertensive activity due to its content of flavonoids and phenols. In another study, biologically active compounds were found in *Pulicaria jaubertii*, including monoterpenoids, sesquiterpenoids, diterpenoids, flavonoids, and phenols [17]. However, the methanolic extract of *Pulicaria jaubertii* has antioxidant, antifungal, and antibacterial activities [19]. The results of these studies may further support the supplementation potential of *Pulicaria jaubertii* for future applications in broiler diets.

To date, there have been no studies on the use of *Pulicaria jaubertii* in broiler diets. However, based on this plant’s previously studied biologically active compounds, it could have a positive effect as a growth promoter and on gut health by modulating the immune response and microbiota of broilers. Therefore, this study aimed to investigate *Pulicaria jaubertii* powder’s effects as a whole plant on the performance, blood biochemistry, internal organs, immune response-related gene expression, and microbiota of broiler chickens.

## 2. Materials and Methods

The whole plant (flowers, seeds, leaves, and stems) of *Pulicaria jaubertii* was collected in the valleys of the city of Ibb in the Republic of Yemen. The plant was classified in the herbarium of the College of Science-King Saud University (NO: 24544) and ground into a fine powder for broiler diets.

### 2.1. Ethical Reference

The Scientific Research Ethics Committee (SREC) of King Saud University, Saudi Arabia, approved all the methods and techniques used in this study (Ethical Reference No: KSU-SE-21-47).

### 2.2. Chemical and Bioactive Composition of Pulicaria jaubertii

An approximate analysis of *Pulicaria jaubertii* samples was performed to determine the content of dry matter, crude protein, ash, crude fiber, and total fat according to the methods of the Association of Official Analytical Chemists [20]. Fatty acid profiles were determined after extraction of total fat and analysis by gas chromatography-mass spectrometry (Agilent Technologies, Palo Alto, CA, USA) according to the method of Biesek et al. [21]. Fatty acids were expressed as g/100 g of identified fatty acid methyl esters.

To identify the main bioactive compounds, they were extracted from *Pulicaria jaubertii* using a methanol solution and then analyzed by gas chromatography-mass spectrometry (Agilent Technologies, Palo Alto, CA, USA) according to the method described by Hussein et al. [17]. To identify the different chemicals, their relative retention times were compared with those of the authentic samples, and their peak-to-peak mass spectra were compared with those of the authentic samples and presented as percentages.

### 2.3. Birds, Study Design, and Housing

A total of two hundred and forty (one-day-old) male broiler chickens (Ross 308) were used for this study. All birds were weighed individually (BW = 43.94) and randomly assigned to four diet groups with ten replicates per group (six birds per replicate). The diet groups (T1, T2, T3, and T4) were supplemented with *Pulicaria jaubertii* powder (whole plant) at 0, 3, 6, and 9 g per kg of the basal diet (control group) for 35 days. The basal diet was formulated as a mash according to the nutrient requirements of the Ross 308 Management Guide recommendations (Aviagen, 2019, New York, NY, USA) at three feeding stages: starter stage (1 to 10 days), grower stage (11 to 24 days), and finisher stage (25 to 35 days) (Table 1). All optimal environmental conditions, including temperature, humidity, and lighting, were maintained according to Ross strain guidelines. The study was conducted in a chamber with electrically heated cages, where the temperature and humidity were set at 33 °C and 50% on the first day and then gradually decreased (2 °C/3 days) to 22 °C and 50% after 27 days. The lighting program was offered for 24 h (30–40 lux) until 7 days of age and for 23 h (minimum 20 lux) after 7 days. Feed and water were offered ad libitum to the birds during the study period. All birds were vaccinated against ND, IB (at 5 and 22 days of age), and against IBDV (at 14 days of age) according to the manufacturer’s instructions (Fort Dodge Animal Health, Fort Dodge, IA, USA).

### 2.4. General Performance Evaluation

Live weight and feed intake were measured throughout the study period. Daily weight gain (final live weight-initial live weight/35 days), daily feed intake (feed provided-residual feed/35 days), and feed conversion ratio (daily feed intake/daily weight gain) were calculated according to El-Ratel et al. [22]. In addition, the production efficiency index ((livability × live weight)/(35 days × feed conversion ratio) × 100) and performance index (live weight growth/feed conversion ratio × 100) were calculated according to Goiri et al. [23].

### 2.5. Sampling and Blood Analysis

At 35 days of age, blood samples were collected from 10 birds in each dietary group after 4 h of feed deprivation in tubes without EDTA via brachial vein to measure biochemical parameters. Serum was separated in a centrifuge at 3000× *g* for 20 min. Serum samples were kept frozen at −20 °C until the biochemical parameters were analyzed. Serum biochemical parameters such as total protein, albumin, glucose, triglycerides, total cholesterol, and high-density lipoprotein were analyzed using colorimetric kits (Randox Laboratories Limited, London, UK) with an automated spectrophotometric analyzer (Chem Well, Awareness Technology, Palm City, FL, USA). According to Albaadani et al. [24], serum low-density lipoprotein (triglycerides-high density lipoprotein-(triglycerides/5)) and globulin (total protein-albumin) concentrations were determined.

### 2.6. Internal Organs Indicators

At 35 days of age, 10 birds per dietary group were selected for slaughter. The carcass of the birds was opened. All internal organs such as the thymus, bursa, spleen, liver, heart, pancreas, and kidneys, were weighed and then calculated as percentage of live weight [25].

### 2.7. Sampling and Gene Expression

One centimeter of jejunum (middle) tissue was collected (35 days old). All tissues from each dietary treatment were washed directly (phosphate-buffered saline), placed in a sterile collection tube containing RNA later solution (Qiagen, Hilden, Germany), and snap-frozen and stored at −80 °C for later quantification of gene expression. Total mRNA was extracted using an mRNA extraction kit (Quick-RNA Miniprep, Zymo Research, Irvine, CA, USA). The quality and quantity of mRNA were checked using a Nanodrop spectrophotometer (Thermo Scientific, NANODROP 2000, Waltham, MA, USA). The mRNA was converted to complementary DNA using the Reverse Transcription Kit (Applied Biosystems, Thermo Fisher Scientific, Oxford, UK) through a PCR system (BIO-RAD, T100 Thermal Cycler, Singapore, Singapore). Gene expression of the cDNA samples was performed by real-time quantitative PCR (7300 Real-Time PCR System, Applied Biosystems, Foster City, CA, USA), using primers to determine the expression of *TNF-α*, *IL-1β*, *IL-4*, *IL-6*, *IL-2*, *IL-10*, *INF-Y*, *sIgA*, *MUC-2*, and *β-actin* genes (Table 2; by Basic Local Alignment Search Tool) and addition of SYBR Green PCR Master Mix (Applied Biosystems, Thermo Fisher Scientific, Foster, CA, USA). The fold change in gene expression for each target gene was calculated using the 2^−ΔΔCt^ method compared to the control group [26].

### 2.8. Cecal Microbiota

Cecal digesta samples were collected from 10 birds from each diet group in a sterile collection tube and stored at −20 °C to determine microbiota colonization [27]. Colonies were clear and easy to count (50 to 300 colonies). A total of 10 μL were grown on specific media for the bacterial species studied. *Lactobacillus* spp. on MRS agar (Himedia, Mumbai, India), *Clostridium perfringens* on BHI agar (Oxoid, Milan, Italy), anaerobic and aerobic bacteria on plate counter agar (Himedia, Mumbai, India), and *Salmonella* spp. and *Escherichia coli* on EMB (Hardy Diagnostics, Santa Maria, CA, USA) were counted using a colony counter. Results were expressed in log10 colony forming units per gram (log_10_ CFU/1 g digesta).

### 2.9. Data Analysis

Statistical analysis of data for all parameters was performed using SAS software [28], based on one-way analysis of variance with a completely randomized design. The following statistical model was used: Observed values (Yij) = data mean (μ) + diet groups (Ti) + random error (eij). Normality of the data (skewness, kurtosis, and boxplot), a statistical difference (*p* < 0.05), and differences between diet group means were examined with the Duncan multiple range test. Means of all parameters for each diet group were expressed as mean ± standard error of the means (SEM).

## 3. Results

The chemical composition and fatty acid profile of *Pulicaria jaubertii* are shown in Table 3. The results of the approximate analysis showed that *Pulicaria jaubertii* (whole plant) was rich in dry matter (92.84%), crude protein (15.52%), ash (17.19%), and crude fiber (35.55%). In addition, the fatty acid profile of *Pulicaria jaubertii* contains saturated fatty acids (50.91%) and unsaturated fatty acids (48.46%) of the total fat (2.80%) in the *Pulicaria jaubertii* plant. Saturated fatty acids such as palmitic acid (20.31%), tridecylic acid (14.91%), and stearic acid (9.59%) and unsaturated fatty acids such as oleic acid (20.48%), linolelaidic acid (14.08%), and linoleic acid (9.31%) were the most abundant residues and represented more than 88.68% of the total fat.

The gas chromatography-mass analysis of the major bioactive compounds in the extract of *Pulicaria jaubertii* is shown in Table 4. The present study revealed that *Pulicaria jaubertii* contains 22 compounds. The phenolic compounds, including benzaldehyde thiosemicarbazone (21.35%) and dimethoxy dimethylsilane (16.35%) were found to be the major constituents and accounted for 37.70% of the bioactive compounds in *Pulicaria jaubertii* (whole plant extract).

The effects of the dietary groups on the overall performance of broiler chickens aged 1 to 35 days are shown in Table 5. The results obtained in the current study for performance indicators such as live weight, daily weight gain, production efficiency index, and performance index were higher when birds were fed different levels of *Pulicaria jaubertii* (T2 to T4) than in the control group (T1; *p* < 0.05). Daily feed intake was not affected by dietary groups (*p* > 0.05), while the feed conversion ratio improved by T2 to T4 compared to T1 (*p* < 0.05).

The effects of the dietary groups on serum biochemical measurements of broiler chickens are shown in Table 6. The diet supplemented with 9 g of *Pulicaria jaubertii*/kg of the basal diet (T4) had higher total protein content compared to T1 and T2 (*p* < 0.05), while albumin, globulin, and albumin to globulin ratio were not affected by the diet groups (*p* > 0.05). The different diet groups also did not significantly affect glucose concentration (*p* > 0.05). Birds fed 9 g of *Pulicaria jaubertii*/kg of the basal diet (T4) had lower triglyceride concentrations compared to T1 and the other groups (*p* < 0.05). Supplemental diet groups (T2 to T4) had lower total cholesterol concentrations (*p* < 0.05), while high-density lipoprotein and low-density lipoprotein were not significantly affected in supplemental diet groups compared to the control group (*p* > 0.05).

The effects of the dietary groups on the relative weight of the internal organs of broiler chickens are shown in Table 7. The birds fed T2 to T4 had a higher relative weight of the liver compared with the control group (*p* < 0.05). The relative weight of the other organs, such as the thymus, bursa, spleen, heart, pancreas, and kidneys, were also not significantly affected among the diet groups compared to the control group (*p* > 0.05).

**Figure 1:**
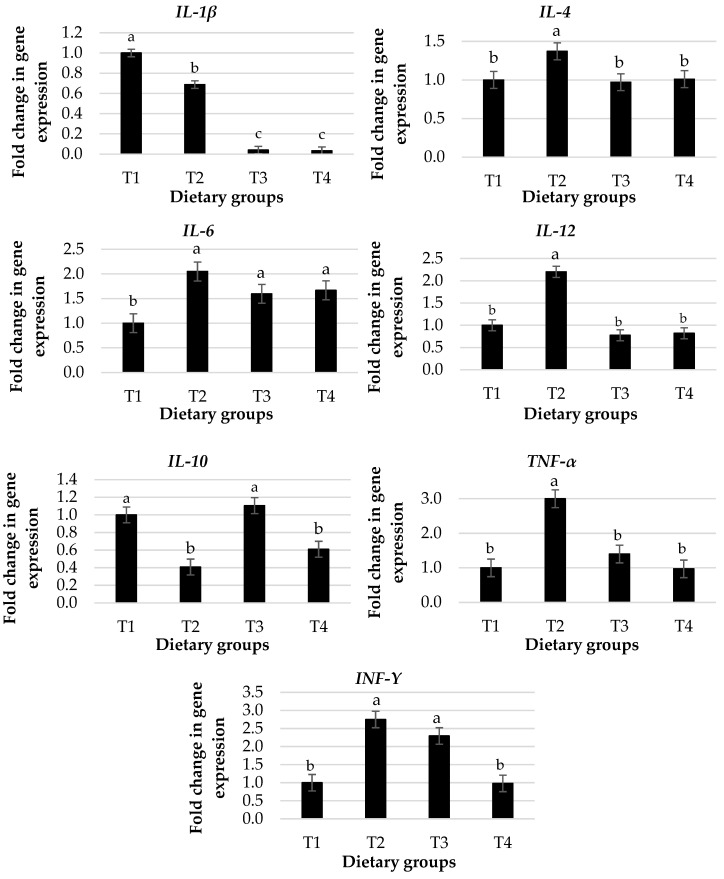
Effect of dietary groups on fold change in gene expression of pre-inflammatory cytokines in the intestines of broiler chickens. ^a–c^ Superscripts above the averages for each parameter within columns express the significant difference (*p* < 0.05). Dietary groups (T1, T2, T3, and T4) were supplemented with *Pulicaria jaubertii* at levels of 0, 3, 6, and 9 g per kg of the basal diet.

The effects of dietary groups on gene expression of pre-inflammatory cytokines in the intestines of broiler chickens are shown in Figure 1. The fold change in the expression of *IL-1β* was decreased in birds fed with diet groups T2 to T4 compared with the control group, whereas *IL-6* was increased (*p* < 0.05). Furthermore, when compared to the other groups, changes in the expression of *IL-4*, *IL-6*, and *TNF-α* were greater in birds fed T2. The change in expression of *INF-Y* was increased in T2 and T3, whereas *IL-10* was decreased in T2 and T4 compared with the control and other groups (*p* < 0.05).

**Figure 2:**
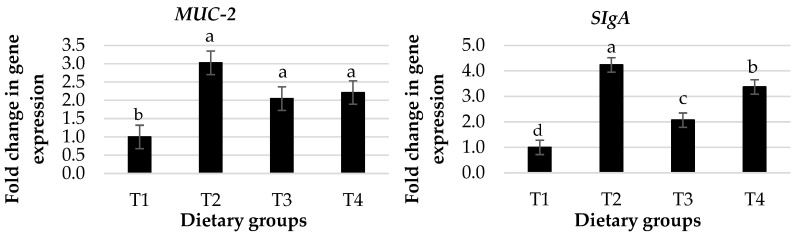
Effect of dietary groups on fold change in gene expression of mucin-2 protein (*MUC-2*) and secretory immunoglobulin A (*SIgA*) in the intestines of broiler chickens. ^a–c^ Superscripts above the averages for each parameter within columns express the significant difference (*p* < 0.05). Dietary groups (T1, T2, T3, and T4) were supplemented with *Pulicaria jaubertii* at levels of 0, 3, 6, and 9 g per kg of the basal diet.

The effects of dietary groups on mucin-2 protein (*MUC-2*) and secretory immunoglobulin A (*SIgA*) gene expression in the intestine of broiler chickens are shown in Figure 2. The current results show that the change in the expression of *MUC-2* and *SIgA* was increased in birds fed with diet groups T2 to T4 compared with the control group (*p* < 0.05). In addition, birds fed T2 had a higher expression of SIgA than T4 and T3.

The effects of the dietary groups on the microbiota in the caecum of broiler chickens are shown in Table 8. Diets supplemented with 3, 6, and 9 g of *Pulicaria jaubertii*/kg of the basal diet (T2 to T4) had a higher quantity of *Lactobacillus* spp. and a lower *Salmonella* spp. compared with the control group (*p* < 0.05). On the other hand, other microbiota in the cecum (anaerobes, *Clostridium perfringens*, aerobes, and *Escherichia coli*) were not significantly affected among the dietary groups compared to the control group (*p* > 0.05).

## 4. Discussion

Renewed interest in the search for new dietary supplements from natural plant sources has gained worldwide attention in recent years due to the development of antibiotic-resistant bacteria [29]. These aromatic and medicinal plants have antimicrobial and anti-inflammatory effects that could be attributed to bioactive compounds (phenols, esters, alcohols, acids, and steroids) that have beneficial effects on broiler production and health [30,31]. The results of these studies could further support the supplementation potential of *Pulicaria jaubertii* for future applications in broiler feeds. In addition, there are no studies on the use of this plant as a feed additive for poultry. *Pulicaria jaubertii* is one of the aromatic and medicinal plants traditionally used to treat a variety of human diseases, including inflammation, colds, diuretics, and digestive disorders [12,13]. The results of the chemical analysis in the current study showed that *Pulicaria jaubertii*, as a whole plant, is rich in crude protein, ash, and crude fiber. In addition, *Pulicaria jaubertii* contains almost equal amounts of saturated and unsaturated fatty acids in the total fat. Saturated fatty acids such as palmitic acid, tridecylic acid, and stearic acid and unsaturated fatty acids such as oleic acid, linoleic acid, and linolenic acid were the most abundant acids. They accounted for more than 88.68% of the total fatty acids. On the other hand, our study by gas chromatography-mass spectrometry revealed that *Pulicaria jaubertii* contained 22 compounds, including benzaldehyde thiosemicarbazone and dimethoxy dimethylsilane as phenolic compounds, which accounted for 37.70% of the bioactive compounds. The results are consistent with several studies that confirmed that the extract of *Pulicaria jaubertii* contains biologically active compounds such as monoterpenoids, sesquiterpenoids, diterpenoids, flavonoids, and phenols [13,14]. Hussein et al. [17] found that the oil extract of *Pulicaria jaubertii* contains 53 volatile components, including sesquiterpene hydrocarbons, monoterpene hydrocarbons, and oxygenated monoterpenes.

Several studies have reported the effect of medicinal plants on broiler chickens [32,33]. In this study, dietary supplementation with *Pulicaria jaubertii* improved the overall performance of broiler chickens, such as live weight, daily weight gain, feed conversion ratio, production efficiency index, and performance index compared to the control group during the study period. Elabd [34] found that dietary supplementation with pulicaria undulata powder as a medicinal plant (3 g/kg diet) gave the best results in the overall growth performance of broilers. This suggests that the effectiveness of dietary supplementation with *Pulicaria jaubertii* on growth performance may be due to the active compounds, fatty acids, and nutrients contained in it. In addition, Dotas et al. [35] hypothesized that the quantity of phytocomponents was responsible for improving the overall performance of broiler chickens. The better overall performance of chickens fed aromatic and medicinal plants such as *Pulicaria jaubertii* might be due to the presence of phenolic compounds and fatty acids, which stimulate the secretion of endogenous intestinal enzymes, thus improving nutrient digestion and gut passage rate [36]. On the other hand, broiler chickens fed medicinal plants may have developed an adaptive response to cope with increased dietary fiber, which ultimately improves digestion and absorption [5]. In addition, higher daily weight gain in broilers fed 3, 6, and 9 g *Pulicaria jaubertii* per kg of the basal diet (T2–T4) without affecting daily feed intake results in better nutrient utilization, as reflected by the improved feed conversion ratio. Production efficiency and performance index are often used as indicators of the production economic status, performance, health, and welfare of broilers [37,38]. Thus, a higher value of production efficiency and performance index indicates that broiler chickens receiving *Pulicaria jaubertii* have consistent body weight gain and are in good health [39]. Dietary supplementation with aromatic and medicinal plants could improve the secretion of intestinal enzymes [40], intestinal morphology [41], and enhance immune function [42] in chickens.

Changes in the serum biochemical profile are metabolic indicators of health and nutritional status [43]. Our study showed that total protein concentration increased in T3 and T4, while total cholesterol concentration decreased due to *Pulicaria jaubertii* and low triglyceride concentration in T4 compared to the control group (T1). Other serum biochemical indicators were also unaffected by the supplemental diet groups. The increase in total protein concentration may indicate that the *Pulicaria jaubertii* levels enhance body protein anabolism in the chickens. The decrease in triglycerides and total cholesterol could be due to disruption of bile acid absorption in the intestine (impairment of bile acid circulation) by *Pulicaria jaubertii* supplementation, resulting in lower serum total cholesterol concentration. In addition, the high fiber content of this plant may play a role in the lowest lipid levels [44]. Another study by Elabd [34] reported that this decrease in total cholesterol concentration could be due to the bioactive compounds of *Pulicaria jaubertii*, which are able to inhibit hepatic 3-hydroxy-3-methylglutaryl coenzyme A activity, an important regulatory enzyme in cholesterol synthesis. The results of our study showed that *Pulicaria jaubertii* had no effect on the relative weight of internal organs (thymus, bursa, spleen, heart, pancreas, and kidneys), while T2 and T3 increased the relative weight of the liver compared to the control group.

Immune cells play a critical role in the secretion of proteins known as cytokines, such as interleukins (ILs), interferon-gamma (IFN-Y), and tumor necrosis factor-alpha (TNF-α), during immune responses by activating and regulating other cells and tissues [45]. Cytokines also serve as the first warning of potential threats to the immune system and are considered a communication point between innate and adaptive responses [46]. The results showed that 3 g of *Pulicaria jaubertii* per kg of the basal diet (T2) induced immune activation and regulation via increased fold changes in the gene expression of pre-inflammatory cytokines such as *IL-4*, *IL-6*, *IL-12*, *TNF-α*, and *INF-Y* and decreased fold changes in the gene expression of *IL-10* compared to the basal diet. This may be due to the beneficial effects of *Pulicaria jaubertii* in increasing immune cell activity and improving gut health. According to Al-Gabr et al. [11] and Mohammed et al. [15], *Pulicaria jaubertii* oil (flower or leaf) showed strong anti-inflammatory activity. *SIgA* is a protein secreted by plasma cells that plays an important role in the gut immune response by preventing antigens from attaching to the epithelium. In addition, *MUC-2* plays a crucial role as the first line of defense, promoting humoral and cellular immunity in the gut [47]. In this study, dietary supplementation with *Pulicaria jaubertii* (T2 to T4) showed increased fold change in *MUC-2* and *SIgA* expression in the intestinal mucosa.

Microbiota have a crucial influence on intestinal development and physiological and immunological functions [48]. A healthy gut is closely related to the balance of intestinal flora [49]. The results of the current study showed that the diet supplemented with *Pulicaria jaubertii* (T2 to T4) had a higher quantity of *Lactobacillus* spp. and a lower *Salmonella* spp. than T1. The antimicrobial activity against *Salmonella* spp. may be attributed to major bioactive compounds, including phenols found in *Pulicaria jaubertii*. *Lactobacillus* spp. is capable of lowering intestinal pH through the secretion of lactic acid, which may lead to the inhibition of *Salmonella* spp. in broiler chickens [50]. Giannenas et al. [41] indicated that phytogenic products rich in phenolic compounds might act against pathogenic intestinal bacteria due to their antibacterial activity, thus promoting the proliferation of beneficial bacteria. The beneficial intestinal bacteria play an important role in protecting the integrity of the intestinal mucosa [51]. Al-Fatimi et al. [52] and Al-Maqtari et al. [14] showed that the extract or oil of *Pulicaria jaubertii* (flower or leaf) has strong antimicrobial activity.

## 5. Conclusions

In conclusion, *Pulicaria jaubertii* (whole plant) contains nutrient compositions, fatty acids, and pharmacologically active components. Therefore, our results suggest that dietary supplementation with *Pulicaria jaubertii* could be used as a natural alternative in broiler diets to improve overall growth performance, production efficiency, and some serum biochemical indicators, as well as to modify the microbiota. In addition, the addition of 3 g *Pulicaria jaubertii*/kg of basal diet resulted in immune activation and regulation through increased gene expression related to the immune response in broilers. Further studies are recommended to investigate the potential mechanism of *Pulicaria jaubertii* in broilers.

## Figures and Tables

**Table 1:** Diet ingredients and calculated nutrient composition of dietary control.

Diet Ingredient (g/kg)	Basal Diet		
	1–10 Days	11–24 Days	25–35 Days
Corn	527	574	616
Soybean meal 48%	391	340	291
Corn oil	37	44	53
Dicalcium phosphate	18	16	15
Limestone	10	9.2	8.6
Salt	4.2	3.2	3.3
DL-Methionine	3.5	3.2	2.9
L-Lysin HCl	2.0	1.9	1.9
L-Threonine	1.3	1.1	0.9
Premix Blank ^a^	5.0	5.0	5.0
Choline Cl 60%	0.9	0.9	1.0
Sodium bicarbonate	0.1	1.5	3.5
Total	1000	1000	1000
Calculated nutrient composition (g/kg)			
ME, kcal/kg	3000	3100	3200
Dry Meter	924.9	932.3	928.3
Crude protein	232.9	211.5	190.9
Crude fat	65.1	72.6	81.6
Crude fiber	28.3	27.2	26.1
Calcium	9.6	8.7	7.9
Non-phytate P	4.8	4.4	4.0
d Lysine	12.8	11.5	10.3
d TSAA	9.5	8.7	8.0
d Threonine	8.6	7.7	6.9
d arginine	14.3	12.8	11.4

^a^ Containing by kg of premix blank: Vit. A: 2,400,000 IU; Vit. D: 1,000,000 IU; Vit. E: 16,000 IU; Vit. K: 800 mg; Vit. B1: 600 mg; Vit. B2: 1600 mg; Vit. B6: 1000 mg; Vit. B12: 6 mg; Biotin: 40 mg; Folic Acid: 400 mg; Niacin: 8000 mg; Pantothenic Acid: 3000 mg; Cobalt: 80 mg; Copper: 2000 mg; Iodine: 400 mg; Iron: 1200 mg; Manganese: 18,000 mg; Selenium: 60 mg; Zinc: 14,000 mg.

**Table 2:** Primer sequences for target genes used in current study by means of real time qPCR.

Target Gene	Primer Sequences (5′ → 3′)	Product Length	Annealing Temperature (°C)	GenBank Accession Number
*TNF-α*	F: GGGAGTGTGAGGGGTATCCT R: CTGCACCTTCTGTCTCGGTT	93	60	MH180383.1
*IL-1β*	F: ACAAGCCGAACAAAGCACAC R: CTCCACATCTGGCTCACGTT	106	61	KY038171.1
*IL-4*	F: GCAGCTGATCCGATTCCTGA R: TCCAACGTACTCTGGTTGGC	99	60	NM_000589.4
*IL-6*	F: GGAGTGGCCAAGAACCAAGA R: ATGCTAAGGCACAGCACACT	109	60	AB028635.1
*IL-10*	F: ACCGTGATTGGCAGGTACAG R: CTGGCCCTGGGTTCACTTAG	123	58	JQ687536.1
*IL-12*	F: ATCCACTGGACCTCAGACCA R: CTCAGAGTCTCGCCTCCTCT	122	59	S82489.1
*INF-Y*	F: TCCCAGAAGCTATCTGAGCAT R: CCACCGTCAGCTACATCTGAAT	200	58	NM_205149.2
*sIgA*	F: TTCCTGAGTTGCCGAGTGAC R: AGGGATTTCTTGCTGGGAGC	106	60	DL232588.1
*MUC-2*	F: CGGTGATGACAACGACTCCA R: AAGTTTGCACAGTCGTTCGC	163	60	AF167707.1
*β-actin*	F: CCTTCCTGGGTAGGTGTCG R: TGGCGTAGAGGTCCTTCCTG	188	60	AJ312193.1

*TNF-α*: Tumor necrosis factor-alpha; *IL*: Interleukin; *INF-Y*: Interferon-gamma; *sIgA*: Secretory Immunoglobulin A; *MUC-2*: Secretory mucin 2. Design and test primers for this sequence using Primer-BLAST.

**Table 3:** Chemical composition and fatty acids profile of *Pulicaria jaubertii*.

**Chemical Composition ^1^**	**g/100 g of *Pulicaria jaubertii***
Dry matter	92.84
Crude protein	15.52
Ash	17.19
Crude fiber	35.55
Total fat	2.80
**Fatty acids profile ^2^**	**g/100 g of total fat**
Caproic acid	2.37
Caprylic acid	1.11
Lauric acid	0.88
Tridecylic acid	14.91
Isomyristic acid	0.55
Myristic acid	0.97
Palmitic acid	20.31
Stearic acid	9.59
Behenic acid	0.22
Saturated fatty acids	50.91
Elaidic acid	0.37
Oleic acid	20.48
Linoleic acid	9.31
γ-Linolenic acid	0.92
Linolelaidic acid	14.08
Arachidonic acid	1.83
Docosatetraenoic acid	0.82
Docosapentaenoic acid	0.65
Unsaturated fatty acids	48.46

^1^ On a dry matter basis, the chemical composition was analyzed in duplicate. ^2^ Using the technique of gas chromatography-mass spectrometry, the fatty acid profile was analysed in duplicate.

**Table 4:** Major bioactive compounds of *Pulicaria jaubertii* extract ^1^.

RT (min)	Hit Name	Mol Formula	%
3.896	Benzeneethanol, 4-hydroxy	C_8_H_10_O_2_	4.24
4.073	2-Methoxyethylsemithiocarbazide	C_4_H_11_N_3_OS	1.59
4.148	1,4-Benzenedicarboxylic acid, methyl ester	C_9_H_7_O_4_	2.49
4.256	Benzaldehyde thiosemicarbazone	C_8_H_9_N_3_S	21.35
4.462	Dimethoxy-dimethyl silane	C_6_H_16_O_4_Si	16.35
4.743	Oxime-methoxy-phenyl	C_8_H_9_NO_2_	1.04
5.098	4′-Hydroxy-3′-methoxyacetophenone	C_9_H_10_O_3_	1.76
6.665	4-Amino-5-(4-acetylphenylazo) benzofurazan	C_12_H_8_N_6_O_3_	2.88
6.74	1,2-Dihydroanthra-thiazole-trione	C_15_H_7_NO_3_S	3.42
7.85	2-Ethylacridine	C_15_H_13_N	2.76
7.947	2-Propen-1, 3-(4-nitrophenyl)-1-phenyl	C_15_H_11_NO_3_	6.86
8.943	Benzoic acid, methyl ester	C_8_H_8_O_2_	1.51
10.327	Benzoic acid, 2,3-bis(trimethylsiloxy), methyl ester	C_14_H_24_O_4_Si_2_	1.78
11.895	Morphinan, 7,8-didehydro-3-methoxy-17-methyl-6-methylene	C_19_H_23_NO	1.55
12.141	Coumarin, 3-(3,4-dimethoxyphenyl)-6-nitro	C_11_H_11_NO_5_	5.72
14.293	Acetic acid, trimethyl ester	C_11_H_29_O_5_PSi_3_	1.34
15.975	Benzophenone-4,4′-dicarboxylic acid dimethyl ester	C_17_H_14_O_5_	5.42
19.38	3-Propenoic acid	C_13_H_19_NO_4_	4.43
22.429	1,4-Benzenedicarboxylic acid, methyl ester	C_9_H_7_O_4_	3.42
25.182	4-Hydroxyphenylacetic acid, ethyl ester	C_16_H_26_O_3_Si	2.84
27.694	Ethyl (2-[(trimethylsilyl) oxy] phenyl) acetate	C_13_H_20_O_3_Si	2.00
29.994	tert-Butyldimethyl (2-propynyloxy) silane	C_9_H_18_OSi	1.15

^1^ The bioactive compounds were analyzed using gas chromatography-mass spectrometry in duplicate.

**Table 5:** Effect of dietary groups on overall performance of broiler chickens from 1 to 35 days.

Parameters	Dietary Groups ^1^				SEM ^2^	*p*-Value
	T1	T2	T3	T4		
Live weight, g	2221.6 ^b^	2347.9 ^a^	2381.6 ^a^	2369.1 ^a^	31.52	0.008
Daily weight gain, g	62.22 ^b^	65.75 ^a^	66.71 ^a^	66.35 ^a^	1.17	0.009
Daily feed intake, g	93.76	89.80	90.93	91.11	1.08	0.129
Feed conversion ratio, g:g	1.51 ^a^	1.36 b	1.36 ^b^	1.37 ^b^	0.01	<0.0001
Production efficiency index	421.6 ^b^	491.3 ^a^	499.6 ^a^	493.1 ^a^	8.88	<0.0001
Performance index	144.6 ^b^	168.5 ^a^	171.4 ^a^	169.2 ^a^	3.09	<0.0001

^a,b^ Superscripts above the averages for each parameter within rows express the significant difference (*p* < 0.05). ^1^ Dietary groups (T1, T2, T3, and T4) were supplemented with *Pulicaria jaubertii* at levels of 0, 3, 6, and 9 g per kg of the basal diet. ^2^ SEM = Standard error of means for groups effect.

**Table 6:** Effect of dietary groups on serum biochemical measurements of broiler chickens.

Parameters	Dietary Groups ^1^				SEM ^2^	*p*-Value
	T1	T2	T3	T4		
Total protein, g/dL	3.68 ^bc^	3.50 ^c^	4.05 ^ab^	4.34 ^a^	0.15	0.002
Albumin, g/dL	1.89	1.66	1.86	2.08	0.09	0.060
Globulin, g/dL	1.78	1.84	2.18	2.26	0.18	0.223
Albumin/Globulin	1.06	0.94	1.01	0.99	0.10	0.869
Glucose, mg/dL	162.70	153.13	162.00	174.50	8.36	0.462
Triglycerides, mg/dL	94.18 ^a^	100.47 ^a^	91.23 ^a^	75.51 ^b^	4.03	0.003
Total cholesterol, mg/dL	136.84 ^a^	121.20 ^b^	108.00 ^c^	122.85 ^b^	3.92	0.0004
High-density lipoprotein, mg/dL	52.64	46.33	46.78	63.00	4.82	0.124
Low-density lipoprotein, mg/dL	65.36	54.76	42.96	44.74	6.26	0.081

^a–c^ Superscripts above the averages for each parameter within rows express the significant difference (*p* < 0.05). ^1^ Dietary groups (T1, T2, T3, and T4) were supplemented with *Pulicaria jaubertii* at levels of 0, 3, 6, and 9 g per kg of the basal diet. ^2^ SEM = Standard error of means for groups effect.

**Table 7:** Effect of dietary groups on relative weight of internal organs of broiler chickens.

Parameters (%)	Dietary Groups ^1^				SEM ^2^	*p*-Value
	T1	T2	T3	T4		
Thymus	0.35	0.39	0.43	0.37	0.05	0.755
Bursa	0.19	0.17	0.20	0.17	0.02	0.419
Spleen	0.09	0.09	0.10	0.11	0.01	0.280
Liver	1.81 ^b^	2.11 ^a^	2.09 ^a^	1.99 ^ab^	0.07	0.030
Heart	0.48	0.46	0.47	0.90	0.19	0.415
Pancreas	0.22	0.21	0.22	0.24	0.01	0.602
Kidney	0.51	0.53	0.39	0.50	0.05	0.194

^a,b^ Superscripts above the averages for each parameter within rows express the significant difference (*p* < 0.05). ^1^ Dietary groups (T1, T2, T3, and T4) were supplemented with *Pulicaria jaubertii* at levels of 0, 3, 6, and 9 g per kg of the basal diet. ^2^ SEM = Standard error of means for groups effect.

**Table 8:** Effect of dietary groups on cecal digesta microbiota (log_10_ CFU/1 g digesta) of broiler chickens.

Parameters	Dietary Groups ^1^				SEM ^2^	*p*-Value
	T1	T2	T3	T4		
Anaerobic bacteria	10.89	12.11	11.91	11.97	0.35	0.082
*Lactobacillus* spp.	9.57 ^b^	10.88 ^a^	11.15 ^a^	11.01 ^a^	0.36	0.021
*Clostridium perfringens*	11.10	10.49	11.01	10.75	0.31	0.523
Aerobic bacteria	11.42	11.89	12.11	11.91	0.28	0.381
*Escherichia coli*	7.86	8.20	7.56	7.66	0.23	0.250
*Salmonella* spp.	8.07 ^a^	7.37 ^b^	6.74 ^c^	6.96 ^bc^	0.19	0.0005

^a–c^ Superscripts above the averages for each parameter within rows express the significant difference (*p* < 0.05). ^1^ Dietary groups (T1, T2, T3, and T4) were supplemented with *Pulicaria jaubertii* at levels of 0, 3, 6, and 9 g per kg of the basal diet. ^2^ SEM = Standard error of means for groups effect.

## Data Availability

The data and analyses presented in this paper are freely available from the corresponding author (H.H.A.-B.).
